# Role of the Death Receptor and Endoplasmic Reticulum Stress Signaling Pathways in Polyphyllin I-Regulated Apoptosis of Human Hepatocellular Carcinoma HepG2 Cells

**DOI:** 10.1155/2018/5241941

**Published:** 2018-12-25

**Authors:** Qihui Luo, Dandan Yang, Qi Qi, Chao Huang, Bing Chen, Wentao Liu, Liangqin Shi, Yu Xia, Li Tang, Jing Fang, Yangping Ou, Yi Geng, Zhengli Chen

**Affiliations:** ^1^Laboratory of Animal Disease Model, College of Veterinary Medicine, Sichuan Agricultural University, Chengdu, Sichuan 611130, China; ^2^Key Laboratory of Animal Disease and Human Health of Sichuan Province, College of Veterinary Medicine, Sichuan Agricultural University, Chengdu, Sichuan 611130, China

## Abstract

Polyphyllin has been reported to exhibit anticancer effects against various types of cancer via the proapoptotic signaling pathway. The aim of the present study was to investigate the role of the endoplasmic reticulum stress and death receptor signaling pathways in PPI-induced apoptosis of human hepatocellular carcinoma HepG2 cells. Analysis demonstrated that PPI could significantly inhibit the proliferation and induce apoptosis of HepG2 cells in a dose- and time-dependent manner. Investigation into the molecular mechanism of PPI indicated that PPI notably mediated ER stress activation via IRE-1 overexpression and activation of the caspase-12 to protect HepG2 cells against apoptosis. In addition, PPI markedly induced the expression of death receptors signaling pathways-associated factors, including tumor necrosis factor (TNF) receptor 1/TNF-*α* and FAS/FASL. Additionally, suppression of the death receptor signaling pathways with a caspase-8 inhibitor, Z-IETD-FMK, revealed an increase in the death rate and apoptotic rate of HepG2 cells. Collectively, the findings of the present study suggested that the ER stress and death receptor signaling pathways were associated with PPI-induced HepG2 cell apoptosis; however, endoplasmic reticulum stress may serve a protective role in this process. The combination of PPI and Z-IETD-FMK may activate necroptosis, which enhances apoptosis. Therefore, the results of the present study may improve understanding regarding the roles of signaling pathways in PPI regulated apoptosis and contribute to the development of novel therapies for the treatment of HCC.

## 1. Introduction

Hepatocellular carcinoma (HCC), the most common type of malignancy of the liver, has been reported as the fifth leading cause of cancer and the third leading cause of cancer associated death worldwide [1]. At present, surgical resection remains to be the preferred method for treating HCC [2]; however, the 5-year survival across all cancer stages is less than 30 % due to postoperative recurrence [3]. Thus, novel therapeutic regimens for the treatment of HCC are urgently required.

**Figure 1:**
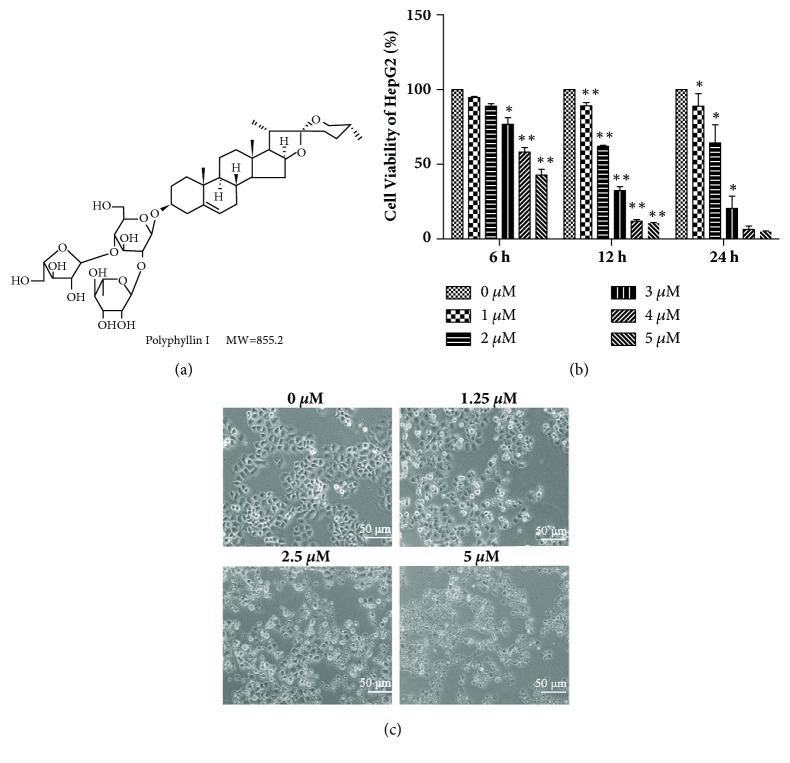
Effects of PPI on the viability of HepG2 cells. Chemical structure of polyphyllin I (a). HepG2 cells were incubated with various concentrations of PPI for 6, 12, and 24 h. Relative cell viability was determined via a Cell Counting Kit-8 assay (b). Cell morphology was observed via microscopy following treatment with PPI at 0-5 *μ*M for 12 h (c). Data were obtained from three independent experiments. ^*∗*^p < 0.05 and ^*∗∗*^p < 0.01, vs. the control group (0*μ*M). PPI, polyphyllin I.

Polyphyllin I (PPI), a steroidal saponin extracted from the rhizoma of* Paris polyphyllin *(Figure 1(a)), has been reported to inhibit the proliferation of various types of cancer, including lung cancer [4], HCC [5], ovarian cancer [6], human osteosarcoma [7], and others. The foremost reported antitumor mechanism underlying the action of PPI was associated with the induction of apoptosis [5]. PPI induced cell apoptosis via activating the caspase cascade, inducing mitochondrial fragmentation [8], and modulating the *β*-catenin signaling pathway [9]. However, extensive investigation of the molecular pathways underlying PPI-induced apoptosis is required to determine the anticancer properties of this particular saponin.

Apoptosis is the process of initiated cell suicide controlled by genes, associated with three main signaling pathways [10]. The endoplasmic reticulum (ER) is the major site in the cell for protein folding and trafficking and is central to many cellular functions [11]. ER stress has been associated with numerous types of cancer and is also a key pathway for the induction of apoptosis. There are two distinct ER stress systems that determine tumor cell survival or cell death [12]. The tumor-adaptive ER stress response mainly occurs via the combination of unfolded proteins with the key ER stress proteins. Glucose-regulated protein (GRP) 78 reduces the accumulation of unfolded proteins to promote tumor cell survival [13]. Prolonged and notable ER stress can activate the apoptosis signaling pathways by inducing the overexpression of DNA damage-inducible transcript 3, also known as GADD153 or CHOP, and promote tumor cell death [14, 15]. The response pathways underlying ER stress mainly involve the protein kinase R-like ER kinase (PERK), activating transcription factor-6 (ATF-6) and endoribonuclease inositol-requiring enzyme 1 (IRE-1)-associated signaling pathways [13]. Previous studies have demonstrated that PPI can induce apoptosis via ER stress in nonsmall cell lung cancer H460 cells [16]. However, whether this occurs in human HCC HepG2 cells is unknown.

In addition, the death receptor signaling pathway is a major contributor to cell apoptosis, which can be triggered via ligands of the tumor necrosis factor (TNF) or Toll superfamily, including TNF ligand superfamily member 10, TNF superfamily member 6 and TNF-*α* [17]. In this signaling pathway, specific ligands bind to their cognate receptors, forming cell death-associated complexes, including the death-inducing signaling complex (DISC), which ensures the oligomerization and activation of pro-caspase-8/10, which leads to subsequent induction of the caspase cascade pathway leading to apoptosis [18]. However, when caspase-8 is inhibited, the TNF receptor 1(TNFR1) signaling network can induce necroptosis, which is driven by receptor-interacting protein kinase (RIPK) 1 and RIPK3 [19]. Necrostatin-1(Nec-1), a specific inhibitor of RIPK1, was reported to potently block RIPK1-dependent necroptosis [20]. Resistance against treatment is a great challenge in the therapy of HCC, particularly against TNFR1/TNF-*α*-mediated apoptosis. Therefore, a treatment that not only induces apoptosis but also has the potency to induce necroptosis may be considered as an effective anticancer agent.

To the best of our knowledge, no studies regarding the possible role of ER stress and death receptor signaling pathways in PPI regulating the apoptosis of HepG2 cells have been conducted. The aim of the present study was to identify the function of ER stress and death receptor signaling pathways. It was determined that ER stress protected HepG2 cells against apoptosis, and the death receptor signaling pathway may also be activated in PPI-induced HepG2 cell apoptosis.

## 2. Materials and Methods

### 2.1. Reagents

PPI was purchased from Chengdu Must Bio-Technology Co., Ltd. (Chengdu, China) and the purity of PPI was ≥ 98%. 4-phenyl butyric acid (4-PBA) was obtained from Sigma-Aldrich (Merck KGaA, Darmstadt, Germany), which was used at a concentration of 20 *μ*M. Nec-1 and caspase-8 inhibitor, Z-IETD-FMK, were purchased from Apexbio Technology LLC (Houston, TX, USA) and used at a concentration of 20 and 10 *μ*M, respectively. Additional reagents employed in the present study were commercially available and of analytical purity.

### 2.2. Cell Culture

The human hepatoma cell line HepG2 was obtained from the Shanghai Institutes of Biological Sciences, Chinese Academy of Sciences (Shanghai, China). HepG2 cells were cultured in Dulbecco's modified Eagle's medium (DMEM; HyClone, GE Healthcare Life Sciences, Logan, UT, USA) supplemented with 10% fetal bovine serum (FBS; Gibco; Thermo Fisher Scientific, Inc., Waltham, MA, USA) and 1% penicillin–streptomycin (Beijing Solarbio Science & Technology Co., Ltd., Beijing, China). Cells were maintained at 37°C in a humidified atmosphere (5% CO_2_).

### 2.3. Cell Viability Assay

PPI was dissolved in dimethyl sulfoxide (DMSO; Sigma-Aldrich; Merck KGaA) as a stock solution, which was stored at -20°C and diluted with medium prior to each experiment. The final concentration of DMSO did not exceed 0.125% during the present study. Cell viability was measured using a Cell Counting Kit-8 (CCK-8; Dojindo Molecular Technologies, Inc., Kumamoto, Japan) assay. Briefly, the cells were seeded at a density of 7x10^3^/well in complete growth medium in 96-well plates. When the cells attained 60 or 70% confluence, different concentrations of PPI were applied to the cells for 6, 12, and 24 h, respectively; CCK-8 was added to each well of the 96-well plate (CCK-8, plus fresh medium at a fixed ratio of 1:10, 100 *μ*l/well), followed by further incubation for 1 h. The absorbance was measured at 450 nm using a microplate reader (Thermo Fisher Scientific, Inc.). The half-maximal inhibitory concentration (IC_50_), defined as the drug dose at which cell growth is inhibited by 50%, was measured using GraphPad Prism software version 5.0 (GraphPad Software, Inc., La Jolla, CA, USA). The following formula was used:

(1) Viability  rate %=absorbance  of  test  sample−absorbance  of  blankabsorbance  of  control−absorbance  of  blank×100%.

### 2.4. Hoechst 33258 Staining Assay

The cells were seeded at a density of 3x10^4^/well in 12-well plates and treated with various concentrations of PPI for 12 h. The cells were washed with phosphate buffered saline (PBS) three times and fixed in 4% paraformaldehyde for 20 min. Following treatment, the cells were washed with PBS three times and stained with Hoechst 33258 (Beijing Solarbio Science & Technology Co., Ltd.) for 30 min. The images of the cells were captured under a fluorescence microscope (Olympus Corporation, Tokyo, Japan). Counted dense stained cells were analyzed in at least 15 visual fields and the apoptosis rate was calculated.

### 2.5. Annexin-V/Propidium Iodide (PI) Double-Staining Assay

An Annexin V-fluorescein isothiocyanate (FITC)-PI Apoptosis Detection Kit (BD Pharmingen; BD Biosciences, Franklin Lakes, NJ, USA) was used to detect apoptosis. HepG2 cells were incubated with PPI or PPI combined with 4-PBA for 12 h. The cells were collected and resuspended in 100 *μ*l binding buffer (1x10^5^cells) with 5 *μ*l Annexin V-FITC and 5 *μ*l PI. The cell suspension was incubated for 15 min at room temperature (20-25°C) in the dark and detected with a FACSCalibur flow cytometer (BD Biosciences) within 1 h of treatment.

### 2.6. Reverse Transcription-Quantitative Polymerase Chain Reaction (RT-qPCR) Assay

HepG2 cells were cultured in 6-well plates (1x10^5^ /well) and incubated with 2.5 *μ*M PPI for 1, 3, 6, and 12 h, respectively. Subsequently, total cellular RNA was extracted with TRIzol reagent (Invitrogen; Thermo Fisher Scientific, Inc.) and the cDNA was synthesized via a PrimeScript™RT Reagent Kit (Takara Biotechnology Co., Ltd., Dalian, China) according to the manufacturer's protocols. Resulting RT products were stored at -80°C until analysis. RT-qPCR was performed in a 12.5 *μ*l mixture containing 1 *μ*l of the cDNA templates, 6.25 *μ*l 2xSYBR Premix Ex Taq II (Takara Biotechnology Co., Ltd.,), and 0.5 *μ*l of the 10 *μ*M upstream and downstream primer; the reaction volume was made up to 12.5 *μ*l with ddH_2_O. The RT-qPCR conditions were as follows: 3 min at 95°C, followed by 40 cycles between 95°C for 10 sec and 58°C for 30 sec, 72°C for 30 sec. The mRNA expression levels of *β*-actin served as a reference and the data were analyzed by Bio-Rad CFX Manager software (Bio-Rad Laboratories, Inc., Hercules, CA, USA). The primer sequences (Sangon Biotech Co., Ltd., Shanghai, China) were listed in Table 1.

### 2.7. Western Blot Analysis

HepG2 cells were treated with PPI (2.5 *μ*M) and the protein was extracted after 3, 6, 12, and 24 h following treatment. The harvested cells were washed with PBS and lysed with radioimmunoprecipitation assay lysis buffer (Wuhan Boster Biological Technology, Ltd., Wuhan, China) to obtain total cellular protein. The protein concentration was determined with a BCA Protein Assay Kit (Wuhan Boster Biological Technology, Ltd.,). In order to detect the levels of protein expression, the protein samples were separated by 10% SDS-PAGE and transferred onto a poly-vinylidene fluoride membrane via a Bio-Rad II System (Bio-Rad Laboratories, Inc.). The membrane was blocked with 5% skim milk powder at room temperature for 1 h and then incubated with primary antibodies at 4°C overnight. The primary antibodies included B-cell lymphoma 2 (Bcl-2), Bcl-2-associated X (Bax), IRE-1, FAS associated via death domain (FADD), FAS, *β*-actin (Wuhan Boster Biological Technology, Ltd.,), TNF-*α*, CHOP and caspase-12 (BIOSS, Beijing, China) at 1:500 dilution and Cleaved-caspase3 (Cell Signaling Technology, Inc., Danvers, MA, USA), cleaved-caspase8 (Beyotime Institute of Biotechnology, Shanghai, China) at 1:1 000 dilution. Subsequently, the membranes were washed with Tris-buffered saline with Tween-20 and incubated with secondary antibodies (Wuhan Boster Biological Technology, Ltd.,) at 1:2 000 dilution for 1 h at room temperature. The protein bands were visualized with a ChemiDoc™MP imaging system (Bio-Rad, Laboratories, Inc.). *β*-actin was used as a loading control. The gray value was analyzed by Image-ProPlus software (Media Cybernetics, Inc., Rockville, MD, USA).

### 2.8. Statistical Analysis

Statistical evaluation was conducted using SPSS 19.0 (SPSS, Inc., Chicago, IL, USA) for Windows package software. All data are presented as the mean ± standard deviation. Differences among multiple groups were compared by one-way analysis of variance, and differences between two groups were compared by the Student's t-test. p < 0.05 was considered to indicate a statistically significant difference. 

## 3. Results

### 3.1. PPI Reduces the Cell Viability of HepG2 Cells

The chemical structure of PPI was presented in Figure 1(a). To determine the effects of PPI on cell viability, HepG2 cells were treated with various concentrations of PPI for 6, 12, and 24 h and the inhibition of cell proliferation was detected via a CCK-8 assay. As shown in Figure 1(b), PPI inhibited the proliferation of HepG2 cells in a dose- and time-dependent manner, with IC_50_ values of 4.67 ± 0.31, 2.25 ± 0.05, and 2.12 ± 0.27 *μ*M at 6, 12, and 24 h, respectively. Thus, we chose 2.5*μ*M and 12 h as the concentration and time of the follow-up study. The morphological changes of HepG2 cells following PPI treatment were observed via microscopy. As shown in Figure 1(c), HepG2 cells treated with PPI were smaller, and cell junctions were not visible.

### 3.2. PPI Significantly Induces HepG2 Cell Apoptosis

**Figure 2:**
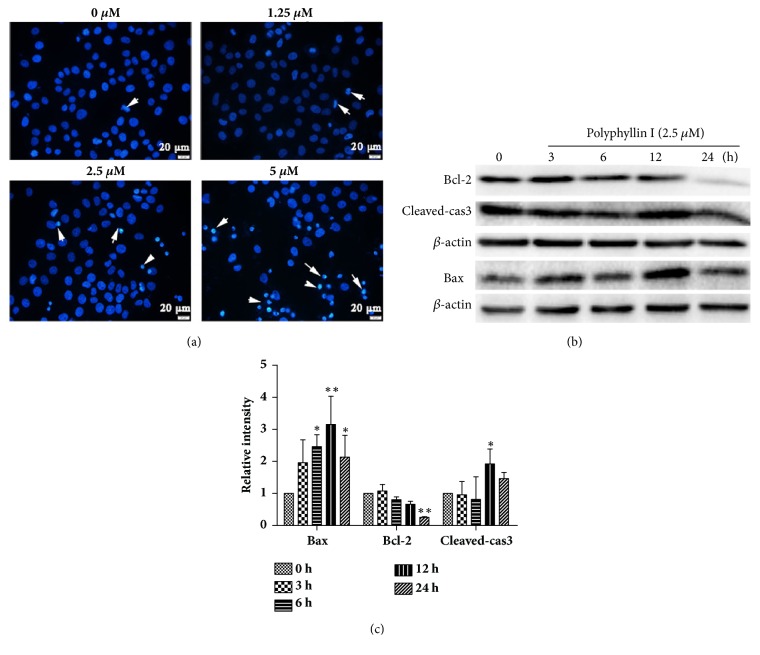
Detection of PPI-induced cell apoptosis. Images of Hoechst 33258 staining were observed by fluorescence microscopy following treatment with PPI (1.25, 2.5, and 5 *μ*M) for 12 h (magnification, x400), white arrows indicate apoptotic cells (a). HepG2 cells were incubated with PPI (2.5 *μ*M) for 0, 3, 6, 12, and 24 h. The expression levels of apoptosis-associated proteins in HepG2 cells were detected by western blotting (b). The alterations in the expression levels of Bcl-2, Bax, and cleaved-caspase3 were statistically analyzed (c). Data were obtained from three independent experiments. The results were presented as the mean ± standard deviation. ^*∗*^p < 0.05 and ^*∗∗*^p < 0.01, vs. the control group (0 h). Bcl-2, B-cell lymphoma 2; Bax, Bcl-2-associated X; PPI, polyphyllin I.

To determine whether the cytotoxicity of PPI against HepG2 cells induces apoptosis, the present study analyzed nuclear morphological changes and the expression of apoptosis-associated proteins following 12 h of treatment with PPI. As shown in Figure 2(a), the HepG2 cells exhibited apoptotic characteristics after treatment with PPI, including karyopyknosis and nuclear fragmentation, cells were notably stained with blue via Hoechst 33258 staining, and viable cells exhibited diffuse and uniform staining. Additionally, the present study analyzed the expression of numerous key proteins involved in apoptosis. Western blotting indicated that PPI downregulated the expression levels of the antiapoptotic protein Bcl-2, and upregulated the expression levels of proapoptotic proteins Bax and cleaved-caspase3 (Figures 2(b) and 2(c)).

### 3.3. PPI Induces ER Stress in HepG2 Cells

**Figure 3:**
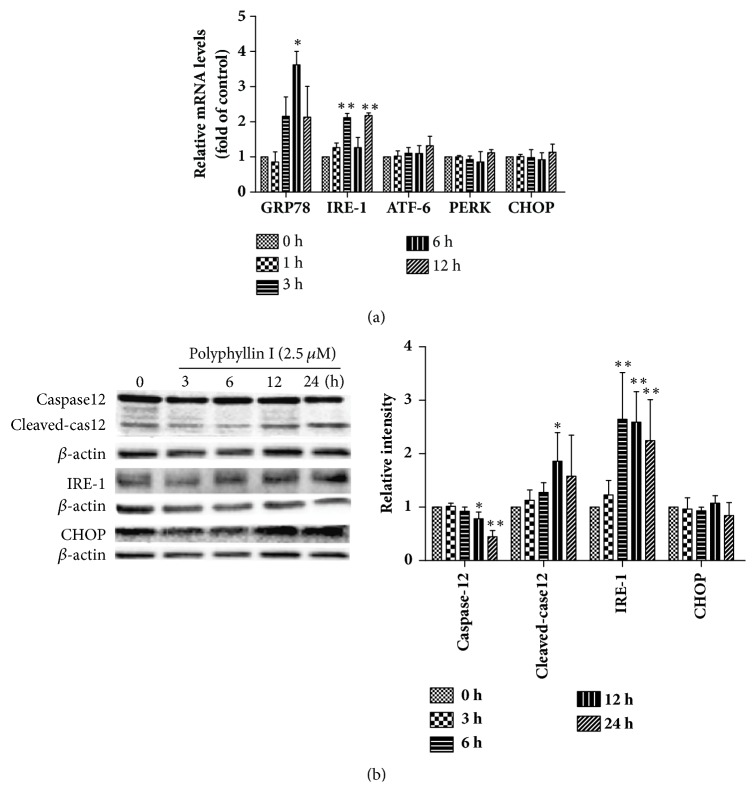
PPI induces apoptosis via the activation of ER stress. HepG2 cells were treated with PPI (2.5 *μ*M) for 1, 3, 6, and 12 h. Total RNA was extracted and synthesized into cDNA, and reverse transcription-quantitative polymerase chain reaction was conducted (a). HepG2 cells were incubated with PPI (2.5 *μ*M) for 0, 3, 6, 12, and 24 h, the expression levels of ER stress-associated proteins were examined by western blot analysis (b). The experiments were repeated three times. The results were presented as the mean ± standard deviation. ^*∗*^p < 0.05 and ^*∗∗*^p < 0.01, vs. the control group (0 h). ER, endoplasmic reticulum; PPI, polyphyllin I.

ER stress serves a key role in the regulation of tumorigenesis and apoptosis. The present study determined the mRNA and protein expression levels of ER stress-associated genes. RT-qPCR demonstrated that the expression levels of ATF-6, PERK, and CHOP mRNA exhibited no notable changes; however, the expression levels of GRP78 and IRE-1 mRNA were significantly increased (Figure 3(a)). Whether PPI specifically activates the IRE-1 signaling pathway was investigated in the present study. Western blot analysis revealed that PPI could upregulate the expression levels of IRE-1 and cleaved-caspase12; no significant differences were observed in the expression levels of CHOP (P > 0.05), a key apoptotic protein involved in the ER stress signaling pathway (Figure 3(b)).

### 3.4. ER Stress Protects HepG2 Cells against PPI-Induced Apoptotic Cell Death

**Figure 4:**
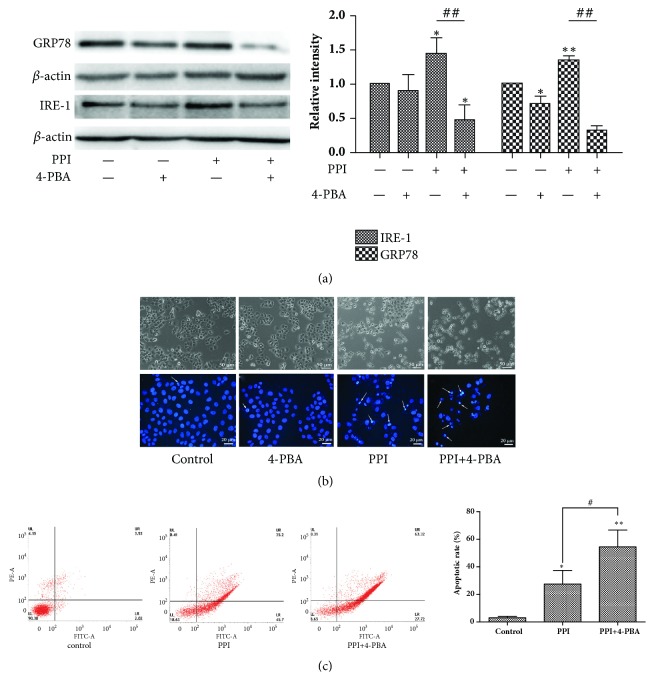
ER stress inhibitor promotes cell death. HepG2 cells were pretreated with 4-PBA (20 *μ*M) for 2 h, followed by incubation with or without PPI(2.5*μ*M) for 12 h. Analysis of IRE-1 and GRP78 protein expression was conducted via western blotting (a). Cell morphology was observed via microscopy (magnification, x100) and cells stained with Hoechst 33258 were observed under a fluorescence microscope (magnification, x400), white arrows indicated the apoptotic cell (b). Apoptotic cells were detected by flow cytometry (c). The experiments were repeated three times. The results were presented as the mean ± standard deviation. ^*∗*^p < 0.05 and ^*∗∗*^p < 0.01, vs. the negative control group; ^##^p < 0.01 vs. 2.5 *μ*M PPI group. ER, endoplasmic reticulum; IRE-1, endoribonuclease inositol-requiring enzyme 1; PPI, polyphyllin I.

The present study examined the apoptosis of HepG2 cells in the presence and absence of the ER stress inhibitor, 4-PBA. Western blotting demonstrated that the protein expression levels of IRE-1 and GRP78 were significantly decreased following treatment with 4-PBA, which indicated that 4-PBA successfully suppressed ER stress (Figure 4(a)). In addition, Hoechst 33258 staining and flow cytometry analysis revealed that the number of apoptotic cells and the rate of apoptosis were significantly increased following treatment with 4-PBA (Figures 4(b) and 4(c)), indicating that ER stress served a protective role against PPI-induced HepG2 cell apoptosis.

### 3.5. PPI Activates the Death Receptor Signaling Pathway in HepG2 Cells

**Figure 5:**
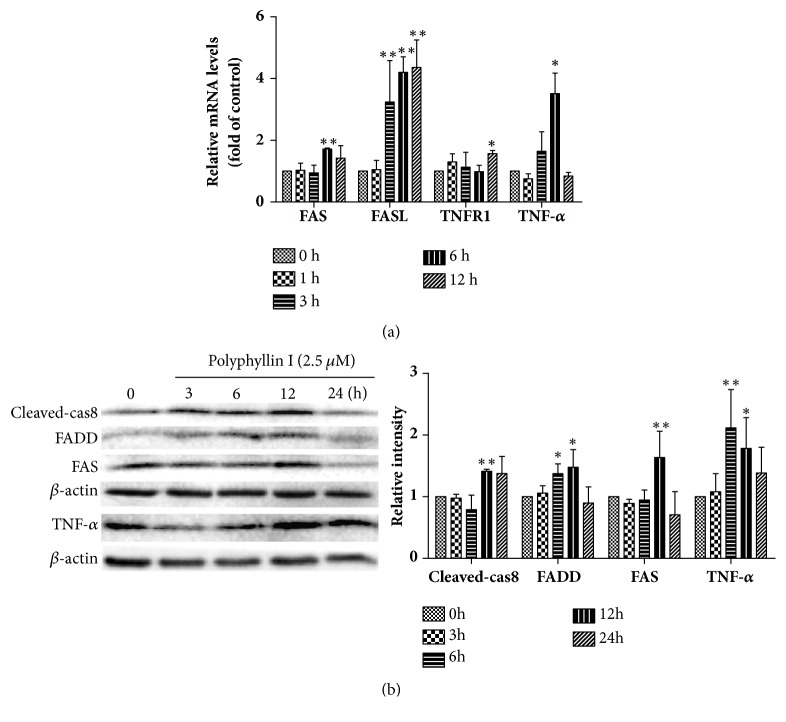
PPI induces apoptosis via the activation of death receptor pathway. HepG2 cells were treated with PPI (2.5 *μ*M) for 0, 1, 3, 6, and 12 h. Total RNA was extracted and synthesized into cDNA, and reverse transcription-quantitative polymerase chain reaction was conducted (a). HepG2 cells were incubated with PPI (2.5 *μ*M) for 0, 3, 6, 12, and 24 h, and the key proteins of the death receptor signaling pathway were examined by western blotting analysis (b). The experiments were repeated three times. The results were presented as the mean ± standard deviation. ^*∗*^p < 0.05 and ^*∗∗*^p < 0.01, vs. the control group (0 h). PPI, polyphyllin I.

The death receptor signaling pathway is a key regulator of apoptosis, cell growth, and proliferation. The present study investigated the expression of key markers of the death receptor signaling pathway, including FAS, FASL, TNFR1, and TNF-*α*. RT-qPCR demonstrated that the expression levels of FAS, FASL, TNFR1, and TNF-*α* mRNA were significantly increased (Figure 5(a)). Western blotting revealed that FAS, TNF-*α*, FADD, and cleaved-caspase8 proteins were significantly upregulated (Figure 5(b)).

### 3.6. Caspase8 Inhibitor Z-IETD-FMK Enhances Cell Death

**Figure 6:**
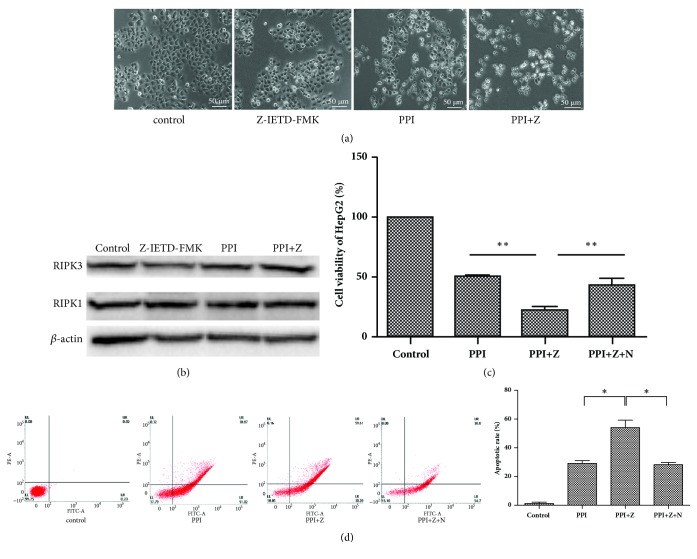
Z-IETD-FMK accelerates the PPI-induced cell death of HepG2 cells.

The present study examined the cell death and expression of necroptosis-associated proteins in HepG2 cells in the presence or absence of Z-IETD-FMK. As shown in Figure 6(a), HepG2 cells were smaller, and the cell junctions were not visible following treatment with Z-IETD-FMK. Additionally, western blotting demonstrated that the expression levels of necroptosis-associated proteins, including RIPK1 and RIPK3, were high; however, no notable alterations between the groups were observed (Figure 6(b)). In the present study, HepG2 cells were incubated with the necroptosis inhibitor, Nec-1. As shown in Figure 6(c), cell viability decreased significantly following pretreatment with Z-IETD-FMK; Nec-1 was observed to protect HepG2 cells against PPI- and Z-IETD-FMK-induced cell death. As shown in Figure 6(d), the cell apoptotic rate of HepG2 cells was significantly increased following pretreatment with Z-IETD-FMK, while the Nec-1 could weaken cell apoptosis induced by PPI- and Z-IETD-FMK.

HepG2 cells were pretreated with Z-IETD-FMK (10 *μ*M) for 2 h or simultaneously pretreated with Z-IETD-FMK and Necrostatin-1 (20 *μ*M) for 2 h. Cell morphology was observed via microscopy following pretreatment with Z-IETD-FMK for 2 h, followed by incubation with PPI (2.5*μ*M) for 12 h (magnification, x100) (a). Expression levels of necroptosis-associated proteins, including RIPK1 and RIPK3, were determined by western blotting (b). HepG2 cells were pretreated with Z-IETD-FMK (Z) and Necrostatin-1 (N), and relative cell viability was determined by a Cell Counting Kit-8 assay (c). The experiments were repeated three times. Apoptotic cells were detected by flow cytometry (d). The results were presented as the mean ±standard deviation.^*∗*^p < 0.05 vs. the negative control group. PIPK1, receptor-interacting protein kinase; Necrostatin-1, RIPK1 inhibitor; Z-IETD-FMK, caspases 8 inhibitor.

## 4. Discussion

Previous studies have demonstrated that traditional Chinese medicines, including PPI, may be considered as potential therapeutic agents for the treatment of human cancers [29]; however, the molecular mechanisms underlying the anticancer properties of PPI, particularly towards HCC, require further investigation. Based on the reported potential that PPI possesses regarding the inhibition of HCC, the present study investigated the properties of PPI. In the present study, PPI was reported to exhibit significant cytotoxicity and inhibition of proliferation in HepG2 cells; a time- and dose-dependent association was also observed. In addition, Hoechst 33258 staining and quantitative determination revealed morphological changes, which were associated with PPI-induced apoptosis. Mechanistically, the results of the present study indicated that the activation of proapoptotic Bax and caspase-3 proteins and reductions of antiapoptotic Bcl-2 protein were associated with the anticancer properties of PPI. The results were consistent with other studies [5, 30], which suggested that induced apoptosis may be an underlying mechanism of PPI in the treatment of liver cancer.

To adapt to endogenous or exogenous stimuli in the tumor microenvironment, cancer cells usually overexpress tumor-adaptive ER stress-associated genes, including ATF6 and GRP78, and simultaneously suppress the expression CHOP associated with tumor-suppressive ER stress [31]. Therefore, the selective induction of tumor-suppressive ER stress responses or the suppression of tumor-adaptive ER stress responses may be considered as potential therapeutic strategies for the treatment of HCC. In the present study, it was demonstrated that PPI selectively upregulated the expression of tumor-adaptive ER stress-associated genes, including GRP78 and IRE-1, and activated caspase-12 protein, promoting the survival of HepG2 cells; no significant differences were observed in the expression levels of CHOP. In further study, 4-PBA was used to confirm the role of ER stress. Interestingly, PPI not only decreased cell viability, but also increased PPI-induced apoptosis following pretreatment with 4-PBA for 2 h, indicating that ER stress served a protective role in PPI-induced apoptosis.

The activation of the death receptor signaling pathway involves the binding of receptors with cognate ligands (TNF-*α* and Apolipoprotein-1), which results in the formation of DISC, consisting FADD and caspase-8. This leads to the activation of pro-caspase8 and downstream caspases [32–34]. In the present study, it was demonstrated that the expression levels of TNF-*α*, TNFR1, FAS, and FASL mRNA were significantly increased. The expression levels of TNF-*α* and FAS proteins also were significantly increased, which were proposed as target proteins for the binding of FADD. Our further study revealed that PPI could upregulate the protein expression levels of FADD and cleaved-caspase8. In addition, the Z-IETD-FMK, an inhibitor of caspase-8, was used to assess the role of death receptor signaling pathway. Numerous cells exhibit significantly increased cell viability following pretreatment with Z-IETD-FMK [35, 36]; however, in the model of PPI-induced HepG2 cell apoptosis, cell viability was significantly decreased. Thus, we speculated that the inhibition of caspase-8 may lead to the failure of RIPK1 and RIPK3 to pyrolyze, which helps death signaling to continue to transmit downstream, accordingly inducing necroptosis. The results of the present study revealed that the cell viability increased significantly following pretreatment with Z-IETD-FMK and Nec-1 compared with Z-IETD-FMK pretreatment alone. Although the protein expression levels of RIPK1 and RIPK3 did not exhibit significant differences across the various treatment groups, indicating that HepG2 cells possess the biological conditions for necroptosis to occur; however, the underlying mechanism of PPI-induced necroptosis in HepG2 cells requires further investigation.

In summary, the ER stress and death receptor signaling pathways were determined to be involved in the PPI-induced apoptosis of HepG2 cells. ER stress was reported to serve a protective role and the inhibition of caspase-8 activated necroptosis. The results of present study revealed that ER stress and caspase-8 exhibit negative regulatory roles in the death of HepG2 cells. Thus, PPI and inhibitor agents may be combined for the effective treatment of HCC in the clinic, which enhance the cell death and elimination of cancer.

## Figures and Tables

**Table 1:** Primer sequences and Tm for RT-qPCR.

Gene	Primer sequence (5′→3′)	*T*_m_	reference
GRP78	F- TAGCGTATGGTGCTGCTGTC R- TGACACCTCCCACAGTTTCA	*T*_m_=60°C	[21]
IRE-1	F- TGGATCCAAAACTACGCCTCC R- GGTCAGATAGCGCAGGGTCTC	*T*_m_=58°C	[22]
ATF-6	F- TATCAGTTTACAACCTGCACCCACTA R- GCAAGGACTGGCTGAGCAGA	*T*_m_=58°C	[22]
PERK	F- TTGTCGCCAATGGGATAG R- CAGTCAGCAACCGAAACC	*T*_m_=58°C	[23]
CHOP	F- CAAAATCAGAGCTGGAACCTGAG R- AGACCTTTCCTTTTGTCTACTCCAA	*T*_m_=60°C	[24]
TNFR1	F- TCCTTCACCGCTTCAGAAAA R- GGGATAAAAGGCAAAGACCAA	*T*_m_=54°C	[25]
TNF-*α*	F- CCCAGGGACCTCTCTCTAATC R- GCTACAGGCTTGTCACTCGG	*T*_m_=58°C	[26]
FAS	F- TCTGGTTCTTACGTCTGTTGC R- CTGTGCAGTCCCTAGCTTTCC	*T*_m_=58°C	[25]
FASL	F- GCAGCCCTTCAATTACCCAT R- CAGAGGTTGGACAGGGAAGAA	*T*_m_=60°C	[27]
*β*-actin	F- TGGCACCCAGCACAATGAA R- CTAAGTCATAGTCCGCCTAGAAGCA	*T*_m_=60°C	[28]

## Data Availability

The data used to support the findings of this study are available from the corresponding author upon request.
